# New York Academy of Medicine—Section on Orthopedic Surgery

**Published:** 1890-03

**Authors:** W. R. Townsend

**Affiliations:** Secretary


### Society Proceedings.

### NEW YORK ACADEMY OF MEDICINE—SECTION ON ORTHOPEDIC SURGERY.

Reported by W. R. TOWNSEND, M. D., Secretary.

STATED MEETING, JANUARY 17, 1890.

V. P. GIBNEY, M. D., Chairman.

Dr. W. R. TOWNSEND presented a case of Congenital Talipes—Right Equino-Varus, and Left Calcaneo-Valgus. The case was of considerable interest, on account of its rarity. Mr. Tamplin states that out of 764 cases where the deformity was congenital, there were only fifteen in which there was varus of one foot and valgus of the other; and only nineteen cases of calcaneus. Dr. Townsend said that this case came to him at the Hospital for Ruptured and Crippled, on December 23d, when only ten days old. It was the mother's second child, and the labor had been normal. There was no history of club-foot in the family. He had already commenced treatment of the right foot, consequently the deformity was not so marked as when he had first seen the case.

Dr. A. M. PHELPS said that this was only the second case of the kind that he had seen; and, in connection with it, he desired to present a plaster cast of two feet removed from the womb of the mother, after her death, at the sixth or seventh month of utero-gestation. It showed equino-varus of the left, and calcaneo-valgus of the right foot, and was an admirable example of the manner in which the deformity had been produced by the pressure of the uterus. There was no history connected with it, beyond what had been stated. The original is in Prof. Volkmann's museum at Halle, Germany.

Dr. JOHN RIDLON remarked that the chief interest in this class of cases is connected with the subject of their causation. He had seen only one other case, which was shortly after the publication of Dr. H. W. Berg's paper on this subject. This patient had the same deformity, and, in addition, clubbed hands on both sides.

Dr. V. P. GIBNEY did not think he had seen more than three or four such cases in an experience of eighteen years. He thought that the retarded rotation theory, as explained by Dr. Berg, accounted very well for these cases.

Dr. A. B. JUDSON said, in regard to the foot affected with calcaneus, that although at first sight it appeared to be a severe deformity, it was quite amenable to treatment, and cited a case published by Dr. Churchill, of Iowa, in support of this assertion, in which he advised simple manipulations, and made an appointment to do a tenotomy one month later. At the end of that time, he was surprised to find that the deformity had entirely disappeared. In another similar case reported by Dr. Prouty, of New Hampshire, the trouble was entirely remedied by the same simple manipulations, so that when the child began to walk, the foot was absolutely normal. A remarkable case had been reported by Dr. Gibney, a few years ago, to the New York Pathological Society, in which the calcaneus was so extreme that the digits had made indentations on the anterior part of the leg.

The paper of the evening on

THE OPERATIVE TREATMENT OF TALIPES CALCANEUS, PARALYTIC, was read by Dr. V. P. GIBNEY, who exhibited eight patients, illustrating the advantages of the operation described in his paper. This operation was that which Mr. Willett, of St. Bartholomew's Hospital, published in the St Bartholomew's Hospital Reports, in 1880. The technique is as follows: A large V-shaped incision over the posterior aspect of the leg, lower fourth, the stem of the V ending at the os calcis,—the stem itself about one and a half inches in length, while each side of the V-shaped portion is about two and a half inches long. The incision exposes the sheath of the tendon. The V-flap is then dissected, the sheath is opened, and the tendo Achillis raised from its bed by a curved director. A strong catgut ligature is passed through the upper portion of the tendon to serve as a means of preventing retraction after section; and the tendon is cut through obliquely, this section being made as oblique as possible. With the vulsellum forceps, each end of the tendon is grasped, and the upper portion pulled down towards the os calcis, while the foot is fully extended and the knee slightly flexed.

The tendon is sutured together with catgut, back and forth, with about three or four heavy sutures; and the end of the V-flap brought down to the end of the stem, and the edges sutured, taking every alternate stitch through the tendon itself.

The aim is to convert the V-shaped wound into a V-shaped cicatrix. It is better to use catgut altogether, in order that the wound may not be disturbed for three or four weeks. Dressings, and plaster-of-Paris, which extends from the toes up to the middle third of the thigh, the knee being flexed to an angle of about 120 degrees, and the foot extended to the full limit, complete the procedure. The operation practised by the reader of the paper differs a little from that of Mr. Willett, in the following particular: Mr. Willett used wire, and exsected a portion of the tendon. The wire he used was merely for fastening the ends of the tendon together. The objections offered to his mode were that the wire cut through the tendon, and that one was in danger of removing too much tendon.

The paper was based upon an analysis of twenty-eight cases operated upon during the past six years. The results showed seventeen good, eight fair, and three poor. The term "good" was defined as a useful foot, without any relapse after a sufficiently long time; ability, also, to walk without a brace or support of any kind. "Fair" was defined as a slight stretching of the cicatrix, but not enough to impair the usefulness of the foot. Shoes with the heel raised and a steel tongue are also required to make the gait satisfactory. "Poor" referred to those cases where the cicatrix had stretched and the deformity had relapsed.

The general results, however, were very satisfactory. The time elapsing between the operation and the date of the last observation was as follows:

| | |
|---|---|
| From three to twelve months, | 9 |
| From one to two years, | 15 |
| From two to three years, | 1 |
| From three to four years, | 1 |
| Five years, | 1 |
| Six years, | 1 |

Sixteen healed by first intention; twelve by granulation. Of those healing by primary union, ten were good, three fair, and three poor. Of those healing by granulation, six were good, five fair, and one poor. In those where granulation took place, the tendon sloughed in three instances, and a portion was removed through the wound. In no instance was a brace required, but particular attention was given to the building of the boot or shoe. The instructions were to have the heel raised at least one inch, to have a stiff counter, and a leather tongue reinforced by tempered steel. The hopelessnes of paralytic calcaneus was discussed at length; the difficulty of correcting the deformity by means of apparatus; the great strain on the spring itself; the frequency of breakages; and the satisfactory results generally.

Dr. JOSEPH D. BRYANT said that he had been especially interested in the statement regarding the changes, which in many cases occur in the length of the new tissues, which had been connected by the operation with the tendo Achillis. The subject was of much importance as bearing upon the question of the behavior of cicatricial tissue elsewhere in connection with the repair of deformities of another kind; and although it does not follow that, because fibrous tissue in this particular situation retracts after the force has been taken from it, that fibrous tissue will do the same thing elsewhere, the subject becomes of immense practical importance in connection with the recent methods for the radical cure of hernia. If we study the behavior of the cicatricial tissue of burns when put on the stretch, we shall find that it will stretch, but that when released, it will return to its former position, or even become more contracted. Such tissue might properly be compared to rubber, which is tireless, while the tissue concerned in the operation under discussion, might be looked upon as rubber which has become tired.

He would like to know if one of the cases which showed such extreme loss of power, was likely to be benefited by a repetition of the operation.

Dr. C. A. POWERS was particularly interested in the subject ot tendon suture of the hand and wrist, in which he had had a considerable experience. He had become convinced that careful antiseptic suture of these cases with proper rest of the parts, yielded uniformly good results. Primary union seemed to be a requisite for a good functional result in hand and wrist cases; for, when healing took place by granulation, the tendons became caught in the cicatrix and there bound. He would like to know in what proportion of cases the author had secured primary union, and how the result seemed to be modified when healing took place by granulation.

Dr. R. H. SAYRE had noticed that some of the patients exhibited were able to move the heel independently of the long flexor of the great toe, and he supposed that as the paralysis had been only partial, the shortening of the tendon had enabled the weakened mucles to act to better advantage. Such cases ought to be much benefited by the persistent use of massage and galvanism, and they present a much more favorable field for operation than those in which the paralysis is absolute; for, under such circumstances, shortening of the tendon only results in the formation of an unyielding fibrous cord.

The progress of the deformity, when untreated, must depend largely upon the amount of damage originally done to the spinal cord. He had seen patients with very marked cavus, who, instead of walking on the bottom of the heel, walked upon the posterior portion, which had, in consequence, developed an elastic buffer. He had hesitated to interfere, as such cases do not hold out much hope of improvement, and the gait is much better than the appearance of the foot would lead one to suppose was possible.

As regards treatment, he favored the use of a brace similar to the one described by Dr. Gibney, or with an elastic spring to take the place of the gastrocnemius. Such an appliance will give the patient comfort, and enable him to move about with less of a wooden tread.

The results shown in the cases this evening are exceedingly good, but he was surprised at the amount of stretching which the cicatricial tissue had apparently undergone. The usual plea against tenotomy is that the resulting scar tissue tends to contract and reproduce the deformity. This, he thought, was a mistake; for the tissue obtained after a subcutaneous tenotomy, is not at all comparable to that obtained in an open wound by the process of granulation. There should be no more secondary contraction after a non-suppurative subcutaneous tenotomy than occurs in tissues after aseptic healing by blood-clot. Whatever elongation has occurred in the cases shown this evening, in all probability took place not in the cicatrix, but in the muscular fibres above, the paralyzed muscle being constantly antagonized by a normal muscle, and thus gradually stretched out.

Dr. RIDLON said that one of the patients exhibited had been seen by him last Summer, and he had then strongly favored tenotomy on account of extreme equinus which existed at that time; but he saw that the foot was now in good position.

In the mechanical treatment of this condition, he had been accustomed to employ the apparatus with the "rubber muscle" at the back; but since Bernard Roth, of London, published the description of his brace for drop-toe, with tempered spring at the back of the leg, he had considered that such an instrument, having a spring running from the garter line with a steel plate to the ball of the foot, was much better than those ordinarily in use.

Dr. H. W. BERG was inclined to take a gloomy view of these cases of poliomyelitis; yet he did not consider them entirely beyond help from neurological treatment. Were it conclusively proven that the nervous supply of the posterior group of leg muscles, for instance, is entirely derived from one level of the anterior gray horns in the spinal cord, or from one series of cells in the spinal cord, it is obvious that if these cells have been entirely destroyed, any electrical treatment must of necessity be useless as regards restoring power to the limb. But it has not been proven that the nervous supply is derived in this way, and it is barely possible that a few cells, giving rise to fibres of any one nerve have escaped the inflammation. The number of these nerve, fibres remaining may be so small as to escape notice in an electrical examination, and yet be sufficient to exert an important influence upon the movements of the foot. Hence, if these healthy nerve fibres and muscle fibres to which they are distributed be stimulated by a galvanic current, they will take on a vicarious action under the irritation of the galvanic current, and will cause, even in old cases of poliomyelitis, as he had frequently observed, a decided improvement in the power to extend the foot. In his experience, fully ninety-five per cent. of the cases had been relieved, although none were cured. He did not think that even the most enthusiastic operators claimed that they did more than relieve their cases. A large number would certainly be benefited by the operation described by Dr. Gibney, but any operation including simply the soft tissues, was hardly a philosophical one, and could not be expected to give as good results as one which would fix the bony tissues.

It is evident that in the cases exhibited, the scar tissue has stretched as the children grew older and the weight of the body increased. This result could be postponed, but not everted, by furnishing a support for the foot.

Dr. JUDSON said that the difficulty in walking experienced by these patients, was due to their inability to use the anterior part of the foot, so that the toe cannot be pressed forcibly against the ground; and hence, they walk very much like one having a peg leg, or an amputation of the anterior part of the foot.

It has been stated that the aborigines of this country were in the habit of performing Lisfranc's amputation upon their captives, who were thus able to work in the fields, but were incapable of rapid locomotion towards liberty. A patient affected with talipes calcaneus is in practically the same condition.

The object of the operation described this evening, seems to be to restore some of this function of the anterior part of the foot, so that the patient, in walking, can bring the weight first on the heel and then on the toe; but it is not easy to understand how the operation can accomplish this, for it is essential that there be very firm union between the calcaneus and the upper extremity of the tibia along the line of the gastrocnemius. With one exception, the cases exhibited could not put their weight on the toe at the same time that the well foot was raised from the ground; nor is it reasonable to suppose that they will retain for any great length of time the slight connection between these parts. He was inclined to think that a cicatrix resulting from primary union, was less liable to contract than one which occurs after a long process of granulation. It is difficult to over-estimate the strain which falls on the tendo Achillis. The great mass of the muscles of the calf gives an indication of this force. The foot may be considered as a lever of the second class, the fulcrum being at the toe, the weight at the ankle, and the power at the heel. The long and short arms of the lever are represented respectively by the portions between the ankle and toe, and the ankle and heel, and the strain produced by the weight of the body is thus multiplied as it falls on the tendo Achillis.

He thought that much could be done for these patients by mechanical treatment, and the object of his brace was to transfer some of the weight of the body to the anterior part of the foot. In the brace formerly described by him, there was a joint at the ankle to arrest motion at a right angle; but the brace has been rendered much more durable and equally efficient by the omission of the joint in the present instrument. The weight, which naturally comes on the plantar surface of the anterior part of the foot in a well person, with this apparatus comes upon the anterior part of the upper portion of the tibia in the neighborhood of its tubercle; so that the patient first strikes the heel, and then puts the weight upon the anterior part of the leg in its upper portion, thereby decidedly improving the gait. The sensation is very much like that of kneeling; for the weight, instead of coming on the ball of the foot as in the healthy person, comes on that part of the tibia which takes the weight when in the kneeling posture.

These cases cannot, of course, be cured by the use of such apparatus; but adult patients are often very glad to wear a simple and durable apparatus which improves the gait.

Dr. Judson remarked that Dr. C. Fayette Taylor had once said that one reason for the muscular degeneration which occurs in these cases, is that the weakened and half paralyzed muscles being compelled to endure such an enormous strain, yield at once; but if they are relieved by means of an apparatus of some of this duty, they are less likely to undergo such degeneration, and, therefore, the chances are better for ultimate improvement.

Dr. Frederick Peterson agreed with the reader of the paper regarding the uselessness practically of the galvanic and faradic currents in these old cases; for, he did not believe that the current could restore destroyed muscle fibre, or degenerated nerve fibres or cells.

Dr. H. L. Taylor said that in considering tenotomy, one must remember that in most cases not only the muscle but the tendon itself is atrophied, so that it is at times a mere thread. These cases of calcaneus are exceedingly difficult to treat, and any real advance will be very welcome; but he considered that the mechanical treatment was fairly satisfactory as a palliative measure. We can retain the foot in a position of election for an indefinite period, and improve locomotion by enabling the patient to transfer the weight from the heel to the ball, not, of course, through the tendo Achillis, but by impinging on the upper end of the tibia by means of an apparatus.

He wished to lay emphasis on the statement that calcaneus could usually be prevented from developing, when these paralytic cases were seen sufficiently early. The foot could be held with absolute precision; and, although he had followed for a considerable time cases of paralysis of the posterior tibial muscles, he could not recall a single one in which calcaneus had developed under proper mechanical treatment.

Dr. Phelps said, that in cases of flail foot, with absolute paralysis, he was accustomed to do an excision, or a Pirogoff's amputation, which is a safe operation, providing firm anchylosis can be secured. Unfortunately this is not always obtainable in children. When the tendon unites primarily, union takes place by blood-clot, and the result is not cicatricial tissue, but a reproduction of the tendon; and, therefore, stretching cannot take place in the tendon itself, but in the body of the muscle. The same argument has been brought forward against the open operation for club foot, only it has been claimed that the cicatricial tissue contracted; but when healing by blood-clot follows that operation, the cicatricial mass does not contract, nor did he believe it yielded.

From birth up to the third or fourth year, and even later, there is a development of the deformity, and, therefore, in estimating the beneficial results from any special method of treatment, one must wait a similar length of time before passing upon the result.

He had been much interested in Dr. Gibney's cases on account of the candor with which they had been presented, and the care exhibited in securing careful histories; but until the ultimate results could be ascertained, he preferred to cut the anterior tendons when required, and apply a brace similar to the one which had been presented; or a brace with a posterior rubber muscle acting on a lever attached to the sole of the shoe; and in special cases, either Pirogoff's amputation or excision.

Dr. Gibney, in closing the discussion, replied seriatim to the questions that had been propounded.

He could not say whether a second operation in one of the cases would be of any benefit.

He had not entirely completed his table of results, and could only say that about one-half of the cases had healed by primary union, and that his analysis, as far as it had gone, failed to show much difference in the results dependent upon the method of healing. He had, of course, always aimed to secure primary union; but some of his best results had been obtained in cases in which the granulating process had been tedious, and even where some of the tendon had protruded and had sloughed away, or had required removal.

He was sorry that he was unable to furnish records of systematic electrical examinations in these cases; but in the hurry of hospital work, this valuable test had frequently been omitted. He had, however, the report of an examination made by Dr. M. A. Starr before the operation on the little boy, who had attracted attention by his ability to stand on his toe and on the ball of the foot. Dr. Starr reported at that time—two years ago—that the posterior group of muscles showed well-marked reaction of degeneration, and failed to respond at all to the faradic current, and he gave it as his opinion that it was very doubtful if recovery would take place. Dr. Gibney thought that most of the gentlemen present would agree with him in saying that the patient now had considerable power in that posterior group of muscles.

In alluding to electrical treatment, he did not intend to disparage all such treatment, but simply to record his own disappointment with it in connection with confirmed cases of calcaneus. He believed with Dr. Berg, that if certain nerve fibres still remained intact, they could be developed by appropriate treatment. He was also willing to admit that an operation which secured anchylosis or synostosis was capable of giving a very useful foot; but from what he had heard of the operation, there seemed to be good cause for doubting the permanency of the results. Besides this, the operation was a much more formidable one than that which he had described in his paper, and it would often be impossible to secure the consent of the parents to perform it, while they would willingly agree to the other operation.

In regard to the mechanical points raised by Dr. Judson, it must be remembered that in addition to the gastrocnemius muscle, the perineal group and some of the interossei are also involved.

In only one of his cases had he met with the ribbon-like form of the tendon, and the result of this case is reported as "poor." When this condition exists, the tendon must be brought farther down, and particular care exercised in the process of suturing, aiming to have the tendon well embedded in the V-shaped flap.

Dr. PHELPS presented a specimen that was apparently an intracapsular fracture of the femur. It had been removed from a man in the dissecting-room, who was noticed to have the legs flexed and abducted, and twenty or more sinuses, healed and unhealed, about the thigh, which had burrowed in every direction. Through a most unfortunate mistake on the part of those who secured the specimen, the soft parts were all carefully removed. The pus is stated to have come from a cavity behind the mass of new bone which is seen in the acetabulum; and the new joint is found to be perfect. When the specimen was exhibited a few evenings since before the Surgical Section, it was thought to be a case of old hip-joint disease; but the specimen clearly shows, since sections have been made, that this is not the case, and is of peculiar interest as illustrating the utter impossibility of curing such a case by mechanical treatment. It was a strictly surgical case, and unless the sinuses were followed up and treated by thorough curetting and free drainage with antiseptic precautions, the man must have died, as he did die, from amyloid disease of the liver and kidneys.

Dr. J. D. BRYANT concurred in the opinion that this was a case of intracapsular fracture.

### A SIMPLE METHOD OF PREVENTING THE BREAKING OF PLASTER AND WAX CASTS.

Dr. PHELPS exhibited two casts so treated. He said that in order to render plaster or wax casts almost unbreakable, it was only necessary to rub well the surface of the cast with plumbago, and then, by the process of electro-deposition, cover the whole surface with a film of copper about 1 mm. in thickness. To illustrate the efficacy of this method, the speaker took one of the specimens, a large cast illustrating Dupuytren's Contraction, and threw it violently upon the floor, without its sustaining the slightest damage.

The other specimen had already been shown at the meeting in connection with Dr. Townsend's case of club feet.

